# Photoferrotrophy: Remains of an Ancient Photosynthesis in Modern Environments

**DOI:** 10.3389/fmicb.2017.00323

**Published:** 2017-03-21

**Authors:** Antonio Camacho, Xavier A. Walter, Antonio Picazo, Jakob Zopfi

**Affiliations:** ^1^Cavanilles Institute for Biodiversity and Evolutionary Biology, University of ValenciaBurjassot, Spain; ^2^Bristol BioEnergy Centre, Bristol Robotics Laboratory, University of the West of EnglandBristol, UK; ^3^Aquatic and Stable Isotope Biogeochemistry, Department of Environmental Sciences, University of BaselBasel, Switzerland

**Keywords:** photoferrotrophy, anoxygenic phototrophs, Archean ocean, ferruginous conditions, iron-rich meromictic lakes, evolution

## Abstract

Photoferrotrophy, the process by which inorganic carbon is fixed into organic matter using light as an energy source and reduced iron [Fe(II)] as an electron donor, has been proposed as one of the oldest photoautotrophic metabolisms on Earth. Under the iron-rich (ferruginous) but sulfide poor conditions dominating the Archean ocean, this type of metabolism could have accounted for most of the primary production in the photic zone. Here we review the current knowledge of biogeochemical, microbial and phylogenetic aspects of photoferrotrophy, and evaluate the ecological significance of this process in ancient and modern environments. From the ferruginous conditions that prevailed during most of the Archean, the ancient ocean evolved toward euxinic (anoxic and sulfide rich) conditions and, finally, much after the advent of oxygenic photosynthesis, to a predominantly oxic environment. Under these new conditions photoferrotrophs lost importance as primary producers, and now photoferrotrophy remains as a vestige of a formerly relevant photosynthetic process. Apart from the geological record and other biogeochemical markers, modern environments resembling the redox conditions of these ancient oceans can offer insights into the past significance of photoferrotrophy and help to explain how this metabolism operated as an important source of organic carbon for the early biosphere. Iron-rich meromictic (permanently stratified) lakes can be considered as modern analogs of the ancient Archean ocean, as they present anoxic ferruginous water columns where light can still be available at the chemocline, thus offering suitable niches for photoferrotrophs. A few bacterial strains of purple bacteria as well as of green sulfur bacteria have been shown to possess photoferrotrophic capacities, and hence, could thrive in these modern Archean ocean analogs. Studies addressing the occurrence and the biogeochemical significance of photoferrotrophy in ferruginous environments have been conducted so far in lakes Matano, Pavin, La Cruz, and the Kabuno Bay of Lake Kivu. To date, only in the latter two lakes a biogeochemical role of photoferrotrophs has been confirmed. In this review we critically summarize the current knowledge on iron-driven photosynthesis, as a remains of ancient Earth biogeochemistry.

## Introduction

Photosynthesis is the main primary production process fueling life on Earth. It requires light as energy source, inorganic carbon to be fixed, and a source of electrons (Hamilton et al., 2016). While oxygenic photosynthesis is currently the dominant process for fixing inorganic carbon into organic matter, this has changed during the history of life (Olson and Blankenship, 2004). Very different conditions existed on early Earth (Canfield et al., 2006), as the chemical environment (e.g., the availability of electron acceptors and donors for biogeochemical processes) and, consequently, the favored biogeochemical processes, differed in the ancient biosphere from those currently prevailing. Even though rates of marine primary production were much lower in these primitive seas than in modern oceans, the most active ecosystems were probably driven by cycling of H_2_ and Fe(II) (Canfield et al., 2006), though other photo- and chemolithoautotrophic processes could also have contributed [e.g., non-photosynthetic Fe(II) and sulfide oxidation]. Still, the early appearance of photosynthesis enhanced biological productivity by orders of magnitude compared to metabolisms based on acetogenesis and methanogenesis as the ancestral forms of carbon and energy metabolisms (Sleep and Bird, 2008; Sousa et al., 2013). Some modern ecosystems (the so-called “analogs,” Burns et al., 2009) still show determinant similar features (i.e., redox conditions, iron and sulfur chemistry) to those predominating on ancient Earth, and offer opportunities to study the processes that sustained microbial life in the primitive biosphere. Photoferrotrophs, which use Fe(II) as an electron donor and light as an energy source for inorganic carbon fixation (Widdel et al., 1993), as well as their biogeochemical role in modern and ancient ferruginous systems, are particularly addressed in this review.

## Evolution of the Biogeochemical Conditions in Ancient Oceans

Once liquid water appeared on Earth around 4.3 Ga ago (Mojzsis et al., 2001), life could emerge and develop in the oceans of the Archean (4 to 2.5 Ga ago) not later than around 3.8 Ga ago (Mojzsis et al., 1996) as evidenced from biologically fractionated organic carbon (Nutman et al., 2016), or even earlier (Abramov and Mojzsis, 2009). The energy required for inorganic carbon fixation was available from sunlight, and from the oxidation of inorganic chemical substrates by chemolithoautotrophs using inorganic electron acceptors. Both photo- and chemolithoautotrophy were, consequently, an option for primary production on the primitive Earth (Canfield et al., 2006). The advent of photosynthesis, however, relieved life from its dependence on the co-occurrence of reduced and oxidized inorganic compounds, as required for chemolithoautotrophy. Indeed, their co-availability is often limited by a high chemical reactivity; i.e., if oxidized and reduced substances readily react chemically, their availability for microbially mediated energy-gaining redox processes diminishes. In this context, light offers the advantage of being an energy source that is not dependent on such co-availability, therefore extending the potential niches for life colonization, although in water columns its availability is also limited to the surface photic layers.

Photosynthesis appeared in the early Archean (Olson, 2006; Fischer et al., 2016), and photosynthetic microbial mats populated benthic environments of oceans shores by around 3.4 Ga ago (Tice and Lowe, 2004), though stromatolites formed around 3.7 Ga ago have also been recently reported (Nutman et al., 2016). It is generally recognized that anoxygenic photosynthesis evolved before the more complex cyanobacterial-type oxygenic photosynthesis (Xiong et al., 2000; Xiong, 2006; Schopf, 2011; Gupta, 2013). The latter is thought to have appeared around 2.75 Ga ago (Buick, 2008), but isotope-based and other data suggest that it may have been present even much earlier (Rosing and Frei, 2004; Planavsky et al., 2014). Some studies, for instance, provide evidence that oxygen was already present in the late Archean (ca. 2.7 Ga) surface environment, supporting oxidative elemental cycling (Anbar et al., 2007; Kendall et al., 2010; Stolper et al., 2010; Crowe et al., 2013; Lalonde and Konhauser, 2015; Frei et al., 2016). These oxygen traces or the consequences of these oxidative processes could have resulted from abiotic reactions such as CO_2_ photodissociation (Lu et al., 2014) and H_2_O_2_ disproportionation (Haqq-Misra et al., 2011), but also from biological processes like benthic oxygenic photosynthesis (Lalonde and Konhauser, 2015). Atmospheric oxygen accumulation, however, did not occur at a global scale before the Great Oxidation Event around 2.33 Ga ago (Bekker et al., 2004; Kaufman et al., 2008; Konhauser et al., 2009; Lyons et al., 2014; Luo et al., 2016). Instead multiple evidences suggest that photoferrotrophy could have been a relevant photoautotrophic process in the ancient biosphere.

**FIGURE 1:**
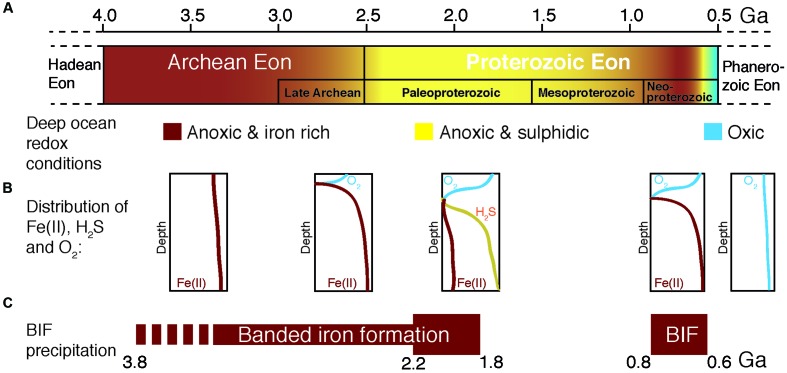
**Geochemical changes from the Archean to the Proterozoic ocean. (A)** Time before present in Giga years (Ga), where color gradients denote postulated changes in deep-sea redox conditions. **(B)** Schematic distribution of reduced iron [Fe(II)], sulfide (H_2_S) and oxygen in the water column of the ocean at each period. **(C)** Periods of banded iron formation (BIF) deposition where the bar width represents the postulated amount of BIF precipitation. Diagram compiled and modified from Anbar and Knoll (2002), Knoll (2003), and Canfield et al. (2008).

**FIGURE 2:**
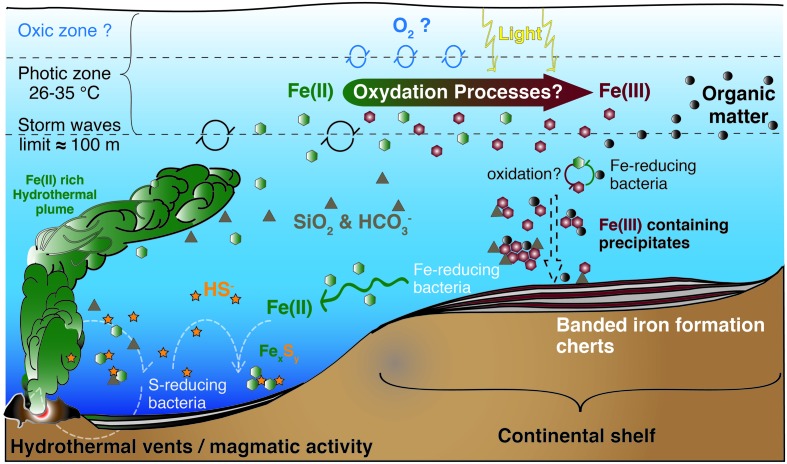
**Model for BIF formation on the continental shelf**. Deep anoxic water, rich in dissolved Fe(II) of hydrothermal origin, is transported onto the continental shelf, where Fe(II) gets oxidized. The produced oxides precipitate from solution toward the seafloor, in association with diverse components such as silica, carbonates or organic matter. The oxidation mechanisms are still unknown and could include a chemical reaction with dissolved O_2_, a UV-light mediated photo-oxidation (less probable), a biological iron-oxidation by anoxygenic photosynthesis using Fe(II) as electron donor, or a combination of the above mentioned processes. Illustration compiled from Konhauser et al. (2002, 2007a), Kappler et al. (2005), Canfield et al. (2006), Severmann et al. (2008), Blake et al. (2010), and Tangalos et al. (2010).

During most of Earth history the ocean was anoxic, and its chemistry was influenced (**Figure 1**) by the activity of microbial life (Blake et al., 2010). Reduced iron [Fe(II)], of hydrothermal origin (Klein, 2005), from biologically processed continental sources (Li et al., 2015), and/or released by tectono-magmatic events (Konhauser et al., 2007a), dominated the mesophilic (Hren et al., 2009; Blake et al., 2010; Poulton and Canfield, 2011), sulfur-poor (Shen et al., 2003; Crowe et al., 2014b) Archean ocean chemistry (**Figures 1**, **2**). From these ferruginous conditions a transition toward a more sulfidic (euxinic) ocean occurred from the late Archean to the Mesoproterozoic (Poulton et al., 2004; Canfield et al., 2008; Reinhard et al., 2009), though sulfide was likely spatially confined to parts of the ocean (Reinhard et al., 2013). Modern analogs of these ancient euxinic environments still exist, such as the Black Sea (e.g., Overmann et al., 1992; Meyer and Kump, 2008), or sulfide-rich meromictic (Noguerola et al., 2016) and holomictic (e.g., Camacho and Vicente, 1998; Camacho et al., 2000, 2001; Camacho, 2006) lacustrine basins. In this sulfide-richer environment of the Proterozoic, sulfide-driven anoxygenic photosynthesis acquired a more relevant role as a primary production process (Kharecha et al., 2005; Johnston et al., 2009). However, ferruginous conditions probably persisted in some zones of the oceans throughout the Proterozoic (Poulton and Canfield, 2011) and even transiently dominated again deep-water chemistry in the Neoproterozoic (Canfield et al., 2008; Planavsky et al., 2009, 2011), though these deep layers were probably aphotic. Later on, during the Phanerozoic (0.54 Ga ago), the ocean became fully oxygenated (Holland, 2006; Hamilton et al., 2016; **Figure 1**). In any case, during the first stages of life, reduced iron dominated ocean chemistry, and microorganisms with iron-based metabolisms were likely important biogeochemical actors on the primitive Earth (Poulton and Canfield, 2011; Kendall et al., 2012; Llirós et al., 2015).

In contrast to euxinic conditions, which can be identified in the geological record by a set of proxies (trace metals, isotopes, and lipid biomarkers), the identification and mineralogical characterization of sedimentary iron enrichments are used to reveal ferruginous deposition conditions (Poulton and Canfield, 2011). Banded iron formations (BIF) are rocks of alternating layers rich *in silica* with layers rich in iron oxides (Posth et al., 2008; Walter, 2011; Frei et al., 2016). BIF occur in geological records of several periods of the Archean (4 Ga to 2.5 Ga) and the Proterozoic (2.5 Ga – 0.54 Ga), especially between the Neoarchean (late Archean) and Early Paleoproterozoic – the Siderian – (2.7–2.4 Ga, Pecoits et al., 2015). Both chemical and biological processes have been proposed as possible mechanisms for BIF genesis in the different time periods (**Figure 2**). These include (Cairns-Smith, 1978; Johnson et al., 2008; Posth et al., 2013): (i) Fe(II) photo-oxidation by UV light (François, 1986; Klein, 2005), or by photochemically produced H_2_O_2_, although the significance of these processes seems rather low (Konhauser et al., 2007a; Pecoits et al., 2015); (ii) chemical oxidation by dissolved oxygen of photosynthetic origin (Holland, 2006; Kump, 2008), and, hypothetically, could even include; (iii) abiotic oxidation of Fe(II) by microbially produced oxidized compounds such as nitrogen oxides (e.g., Klueglein and Kappler, 2013), though the sufficient availability of these oxides to support a visible contribution of this mechanism is yet to be shown. However, (iv) direct oxidation by microbial processes (Garrels et al., 1973; Hartman, 1984; Li et al., 2011; Posth et al., 2013; Czaja et al., 2013; Chan et al., 2016), mainly iron-oxidation by anoxygenic phototrophs using Fe(II) as electron donor, seems an attractive mechanism for early BIF formation, particularly for the time before oxygenic photosynthesis appeared on Earth (Ehrenreich and Widdel, 1994a; Konhauser et al., 2002, 2005, 2007a,b, 2011; Kappler and Newman, 2004; Kappler et al., 2005; Croal et al., 2009; Fru et al., 2013; Li et al., 2013; Eickhoff et al., 2014; Sun et al., 2015).

## Photoferrotrophy and Photoferrotrophs

Iron is the most abundant metal on Earth, and among the most abundant elements in the Earth’s crust (Kendall et al., 2012). Under circumneutral pH conditions Fe(II), particularly in its ionic form (Fe^2+^), it is rapidly oxidized by oxygen to highly insoluble Fe(III) oxides. As a consequence iron can become a limiting nutrient for primary producers in vast areas of the well-oxygenated modern ocean, where iron concentration is typically <1 nM (Martin and Fitzwater, 1988). Under anoxic reduced conditions Fe(II) is the dominant iron form. Ionic Fe^2+^ is several orders of magnitude more soluble than Fe(III) (Stumm and Morgan, 1995; Cornell and Schwertmann, 2003) and can reach high concentrations in ferruginous water columns (**Table 1**) and in the pore water of freshwater sediments (e.g., Bravo et al., 2015). In sulfur-rich environments iron reacts with dissolved sulfide forming highly insoluble iron-sulfide minerals (e.g., Zopfi et al., 2004).

**Table 1:** Some features of the best studied Archean Ocean analogs (Lakes Matano, Kabuno Bay of Lake Kivu, Lake La Cruz and Lake Pavin), as well as of some other iron-rich natural meromictic lakes (mining lakes are excluded).

Environment/ Lake	Location	Surface (km^2^)	Maximum depth (m)	% PAR at the top of the anoxic layer	Max Fe concentration in anoxic waters (μmol l^-1^)	Estimated iron oxidation rate *in situ* (μmol l^-1^ d^-1^)	Phototrophic bacterial abundance	Reference
Archean ocean		nda	nda	Up to 100	40–120	14	nda	Holland, 2004; Canfield, 2005; Kappler et al., 2005; Crowe et al., 2008a; Walter et al., 2014
Kabuno Bay of Lake Kivu	DRC	Aprox. 70	150	1–5	1200	>100	8 × 10^7^ (GSB)	Llirós et al., 2015; Katsev et al., 2017
La Cruz	Spain	0.017	21	0.1	276	0.174–1.396	2.2 × 10^6^ (PSB) 3.1 × 10^6^ (GSB)	Vicente and Miracle, 1988; Rodrigo et al., 2000; Camacho et al., 2003; Walter et al., 2014; Picazo, 2016; Camacho et al., 2017
Matano	Indonesia	164	590	0.1	140	0.034–0.27	2 × 10^5^ (GSB)	Crowe, 2008; Crowe et al., 2008a, 2014a,b
Pavin	France	0.445	92	nda	1200	nda	nda	Bura-Nakic et al., 2009; Busigny et al., 2014
Clouds	USA	0.5	31	nda	nda	nda	nda	Anthony, 1977
Hall	USA	0.03	16.2	nda	1380	nda	nda	Culver, 1977; Balistrieri et al., 1994
Paul	USA	1	12	nda	120	nda	nda	Taillefert and Gaillard, 2002
Nordbytjernet	Norway	0.28	23	nda	710	nda	nda	Hongve, 1997, 1999, Hongve, 1997, 2003
Store Aakalungen	Norway	0.132	32.5	nda	nda	nda	nda	Kjensmo, 1962, 1967
Skjennungen	Norway	0.034	17.8	nda	nda	nda	nda	Kjensmo, 1988
Svetloe	Russia	nda	39	nda	240	nda	nda	Savvichev et al., 2017

*GSB and PSB stand for green and purple sulfur bacteria, respectively; nda, no data available*.

The biological significance of iron is based on its redox behavior, with Fe(III) and Fe(II) as main oxidation states, making it a suitable electron donor or acceptor, respectively, for different dissimilatory processes (Ehrenreich and Widdel, 1994a; Straub et al., 2001, 2004; Lovley et al., 2004; Weber et al., 2006) that greatly influence the iron cycle on Earth. These microbial processes include: (i) anaerobic ferric iron reduction with organic matter and H_2_, perhaps the first respiratory process on Earth (e.g., Vargas et al., 1998; Lovley et al., 2004); (ii) anaerobic nitrate-reducing Fe(II)-oxidation (e.g., Straub et al., 2004; Weber et al., 2006); (iii) aerobic chemolithoautotrophic microorganisms that oxidize Fe(II) with oxygen (e.g., Emerson and Weiss, 2004; Chan et al., 2016) and; (iv) Fe(II) photosynthesis, where photoferroautotrophic microorganisms use light energy (photo-) and the reducing power of Fe(II) (-ferro-) to fix inorganic carbon (-autotrophic), according to Widdel et al. (1993):

$${\mathrm{4Fe}}^{2+}+{\mathrm{HCO}}_{3}^{-}+\mathrm{10}H_{2}O+\mathrm{light}\left(h\upsilon \right)\to \mathrm{4Fe}{\left(\mathrm{OH}\right)}_{3}+({\mathrm{CH}}_{2}O)+{7H}^{+}$$

Anoxygenic photosynthesis with Fe(II) as electron donor using exclusively photosystem I has been proposed as being the earliest type of photosynthetic process (Des Marais, 2000; Xiong et al., 2000; Raymond et al., 2003). While there is currently no supporting evidence from genomic studies (Frigaard and Bryant, 2008; Gupta, 2013) that photoferrotrophy evolved prior to anoxygenic photosynthesis with other electron donors, such as sulfide or hydrogen, the ancient environmental conditions suggest that Fe(II)-photosynthesis was an important metabolic process in the iron-rich Archean oceans, prior to the appearance of oxygenic photosynthesis. Molecular phylogenetic analyses of enzymes involved in bacteriochlorophyll biosynthesis support the existence of anoxygenic phototrophs prior to oxygenic phototrophs (Xiong et al., 2000; Xiong, 2006; Gupta, 2013), although horizontal gene transfer between photosynthetic organisms complicates the phylogeny (Raymond et al., 2003). Before demonstrating its occurrence, photoferrotrophy was hypothesized as a possible autotrophic metabolism for different types of phototrophic prokaryotes (Olson and Blankenship, 2004; Olson, 2006). Some authors suggested the possibility that both cyanobacteria (e.g., Cohen, 1984, 1989; Cohen et al., 1986; Pierson and Olson, 1989; Pierson et al., 1999) and green sulfur bacteria (Garcia-Gil et al., 1990) could perform anoxygenic photosynthesis with Fe(II) as electron donor. In spite of their capacity for anoxygenic photosynthesis (Cohen et al., 1975, 1986), and its crucial role on Earth biogeochemistry, no evidence for the occurrence of photoferrotrophy has been provided so far for modern cyanobacteria (Trouwborst et al., 2007; Swanner et al., 2015; Hamilton et al., 2016).

Anoxygenic photosynthesis using Fe(II) as electron donor was unequivocally demonstrated, for the first time, in purple bacteria, some of which were able to grow either photoautotrophically and/or photoheterotrophically (Widdel et al., 1993; Ehrenreich and Widdel, 1994b). In the meantime, photoferrotrophy has been detected in bacteria of different phylogenetic groups and either from freshwater or marine origin, including representatives of purple sulfur bacteria (PSB, Gammaproteobacteria), purple non-sulfur bacteria (PNSB, Alphaproteobacteria), and green sulfur bacteria (GSB, Chlorobi). Even though most of these anoxygenic phototrophs are able to use several types of electron donors commonly found in anaerobic environments (e.g., H_2_S and H_2_; Bryant and Frigaard, 2006; Camacho, 2009), there are some specific strains that have been demonstrated to perform Fe(II)-dependent anoxygenic photosynthesis (Croal et al., 2009). These include strains phylogenetically related to PSB, such as the freshwater *Thiodictyon* sp. (Croal et al., 2004; Hegler et al., 2008), and the marine species *Rhodovulum iodosum* and *Rhodovulum robiginosum* (Straub et al., 1999; Wu et al., 2014); freshwater PNSB such as *Rhodobacter ferrooxidans* sp. strain SW2 (Ehrenreich and Widdel, 1994b; Hegler et al., 2008), *Rhodomicrobium vannielii* (Heising and Schink, 1998), and *Rhodopseudomonas palustris* (Jiao et al., 2005; Jiao and Newman, 2007); as well as a freshwater species of GSB, *Chlorobium ferrooxidans* (Heising et al., 1999). Although the PNSB *Rhodobacter capsulatus* is also capable of oxidizing Fe(II) in presence of light, this is not a real photoautotrophic process (Caiazza et al., 2007; Kopf and Newman, 2012) and can be considered as a Fe(II) detoxification mechanism (Poulain and Newman, 2009).

The best studied photoferrotrophs are purple non-sulfur bacteria (e.g., Widdel et al., 1993; Eickhoff et al., 2013; Wu et al., 2014). When comparing PSB with PNSB, rates of Fe(II) photooxidation are influenced by the response to light (e.g., light saturation) of each photoferrotrophic species. This was shown by Kappler et al. (2005), comparing the iron photoxidation of the purple sulfur bacteria *Thiodictyon* sp. and the purple non-sulfur bacteria *Rhodobacter ferrooxidans*. *R*. *ferrooxidans*, with lower saturation intensity, is able to efficiently oxidize iron at rates of about 32 pmol Fe(II) h^-1^ cell^-1^ at 20°C, circumneutral pH and under light saturation conditions (Hegler et al., 2008). Photoferrotrophs display mechanisms preventing cell encrustation with oxidized iron (Miot et al., 2009; Saraiva et al., 2012; Wu et al., 2014).

Presumably, photoferrotrophs use a periplasmic c-type cytochrome for cyclic electron flow and other iron-oxidoreductases for linear electron transfer (Bird et al., 2011). In *Rhodopseudomonas palustris*, the *pio* (phototrophic iron oxidation) operon is essential for phototrophic Fe(II) oxidation, as it encodes, apart from a membrane transport protein, for small high-potential redox proteins such as *Pio*C and cytochrome *c*_2_ (Jiao and Newman, 2007). The latter is essential for cyclic electron flow, whereas *Pio*C linearly transfers electrons from iron (Bird et al., 2014). Operons related to phototrophic iron oxidation are also described for other purple non-sulfur bacteria, such as the *foxEYZ* operon from *R. ferrooxidans* SW2 and *R. capsulatus*, also coding for proteins of similar functions as those encoded by the *pio* operon of *R. palustris* (Croal et al., 2007; Saraiva et al., 2012).

Green sulfur bacteria are among the most ancient photosynthetic organisms (Gupta, 2013; Gauger et al., 2016). Although it has been hypothesized that several *Chlorobium* species could perform Fe(II)-dependent anoxygenic photosynthesis in lakes (e.g., Garcia-Gil et al., 1990), *Chlorobium ferrooxidans* (both strains KoFox and KB) is so far the only GSB species whose photoferrotrophic ability has been demonstrated. While all other GSB species can use various sulfur compounds as electron donors, genomic analyses revealed that *C. ferrooxidans* has apparently lost most genes involved in oxidation of sulfur compounds (Frigaard and Bryant, 2008). Heising et al. (1999) showed that strain KoFox was able to grow photoferroautotrophically in co-culture with *Geospirillum* sp. strain KoFum; whose fermentation of fumarate to organic acids enhanced Fe(II) oxidation by KoFox. Among other possible explanations, acidification of the medium by organic acids could be responsible for this enhancement as this could increase the solubility and hence the bioavailability of iron. Growth of photoferrotrophic KoFox is stimulated by the presence of silica, which is possibly due to its influence on iron chemistry, mediating encrustation patterns and cell–mineral interactions and reducing iron toxicity (Posth et al., 2010; Gauger et al., 2016). On the other hand, *Chlorobium ferrooxidans* strain KoFox is able to oxidize Fe(II) at very low light intensities, with saturation at <50 lux compared to saturations of 400 lux for *R. ferrooxidans* and 800 lux for *Thiodictyon* (Hegler et al., 2008). The capacity of GSB to present spectral modifications in their pigments (Borrego et al., 1997; Chew et al., 2007) and grow under a very dim light (e.g., <0.0005% of surface irradiance, Overmann et al., 1992; Manske et al., 2005), enable GSB to thrive in deep zones of anoxic ferruginous basins where Fe(II) is available and light availability is still sufficient for a “frugal” photosynthesis (Ormerod et al., 1993), making these organisms good candidates for performing photoferrotrophy in current Archean ocean analogs.

Although the possibility of manganese-oxidizing photo-synthesis has also been proposed (Olson, 1970), even as a possible previous step to the advent of oxygenic photosynthesis (Johnson et al., 2013; Fischer et al., 2016), so far no phototrophic Mn(II)-oxidizing bacterium has been found and supporting evidences for manganese-oxidizing photosynthesis are “highly tenuous” (Jones and Crowe, 2013). Even chlorinic photosynthesis –biologically mediated photolytic oxidation of aqueous chloride to form halocarbon or dihalogen products, coupled with CO_2_ assimilation-, has been proposed as a potential metabolism on exoplanets under conditions that may approximate to the terrestrial Archean (Haas, 2010).

## Photoferrotrophy in Modern Water Columns: Occurrence and Significance

Apart from the information preserved in the geological record, knowledge about the metabolisms supporting life on ancient Earth, e.g., anoxygenic photosynthesis, can be gained by studying environments that are considered as “modern analogs” of different states of the ancient ocean (Burns et al., 2009). Most meromictic lakes and other permanently stratified water bodies are euxinic, i.e., anoxic and sulfidic, below the chemocline (Zadereev et al., 2017). Sulfidic basins of stratified lakes and closed seas might resemble the sulfidic ocean of the Paleoproterozoic and Mesoproterozoic. Contrastingly, sulfide poor anoxic layers of iron-rich stratified (often meromictic) lakes are the most similar modern environments to the iron-rich Archean oceans, where photoferrotrophy could have played a crucial role in governing the biogeochemistry and providing energy to drive microbial growth and evolution (Llirós et al., 2015). Ferruginous water columns are rare, largely unexplored ecosystems of which only freshwater representatives exist today because of the high sulfate concentrations in the modern ocean. In recent years, a few lakes have been described and studied as possible Archean ocean analogs providing insights on how ancient photoferrotrophs could flourish in the Archean ocean and on their possible role in its biogeochemistry. Koeksoy et al. (2016) reviewed the biogeochemical characteristics of some of these environments and paid special attention to iron chemistry and the usefulness of such systems to interpret Precambrian BIF deposition.

According to the current knowledge, the most appropriate Archean ocean analogs are natural iron-rich meromictic lakes. Meromictic lakes show a permanent stratification whose bottom water (monimolimnion), which has higher concentrations of dissolved salts (Imboden and Wüest, 1995), does not mix with the overlaying waters due to the water density gradient and other factors linked to climate, lake morphometry and water flow (Boehrer et al., 2009). In iron-rich meromictic lakes the high monimolimnetic iron concentrations contribute to the water column stability, which drove Kjensmo (1967) in his seminal manuscripts to use the term “iron-meromixis”. However, the strongest density gradient in these lakes is commonly owed to dissolved compounds other than iron (e.g., calcium bicarbonate, Rodrigo et al., 2001). In stratified iron-rich lakes the interface between oxic and anoxic water bodies, the oxycline, is accompanied by a steep gradient of iron forms, the so-called “ferrocline” (Bravidor et al., 2015). In addition to iron meromictic lakes, temporally stratified lakes have also been proposed as possible study sites as ancient ocean analogs. Specifically, these are the holomictic man-made gravel Lake Vechten in the Netherlands (Koeksoy et al., 2016), as well as dimictic iron-rich Boreal Shield lakes (lakes L227 and L442 from the Experimental Lake Area in Canada) where molecular microbial and stable isotope data suggest that these lakes may be also good candidates to be studied as analogs of ancient oceans (Schiff et al., 2016).

In **Table 1** we summarized the basic characteristics of most of the currently described meromictic ferruginous lakes. Mining lakes were excluded because of the acidic pH and strongly different biogeochemical conditions (Boehrer et al., 2009). The best studied ferruginous systems are the lakes Pavin (France), Matano (Indonesia), La Cruz (Spain) and the Kabuno Bay of Lake Kivu (Democratic Republic of the Congo). Even though most of these lakes show maximum iron concentrations largely overpassing those estimated for the Archean ferruginous oceans (**Table 1**), iron concentrations at the photic anoxic zones of the lakes are lower, likely with higher resemblances to those estimated for the Archean ocean (Holland, 2004; Canfield, 2005; Canfield et al., 2005). However, although it can be stated that iron was present at relatively high concentrations, there is a high uncertainty in the estimation of the iron concentrations in the Archean ocean, so only categorical comparisons can be made with some confidence. On the other hand, some of these iron-rich lakes are also known to accumulate high concentrations of methane and CO_2_ in the bottom waters (Crowe et al., 2011), which might cause catastrophic limnic eruptions, similar to what occurred in the African Lakes Nyos and Lagos (Zhang and Kling, 2006; Katsev et al., 2017).

### Lake Pavin

Lake Pavin is a meromictic circular crater lake in the French Central Range (Massif Central) (Bura-Nakic et al., 2009). Its permanent chemocline starts at 60 m depth and below the anoxic iron-rich monimolimnion extends to the lake bottom. In summer, a thermal stratification additionally occurs (Aeschbach-Hertig et al., 2002).

Although sulfide is detectable in the anoxic waters, the high iron concentrations (maximum of up to 1200 μmol l^-1^) cause most of the sulfide to be in the form of colloidal iron sulfide (Bura-Nakic et al., 2009). Meanwhile, isotope studies showed that most of the iron isotope variability observed in sedimentary pyrite can be tied to water column cycling foremost to the oxidation of dissolved ferrous iron (Busigny et al., 2014). In fact, the sulfur cycle is one of the main active element cycles in this lake with (potentially chemolithoautotrophic) Epsilonproteobacteria (and apparently also non photosynthetic sulfur bacteria) playing a key role in the oxidative phase of the sulfur cycle (Biderre-Petit et al., 2011a). 16S rRNA gene sequences highly resembling the microaerophilic iron oxidizer *Gallionella ferruginea* were also abundantly retrieved in the upper part of the chemocline (Lehours et al., 2007). Interestingly a highly diverse community of unicellular eukaryotes, mainly heterotrophic and mixotrophic microbes that could benefit from the abundant and diverse prokaryotic community (Lehours et al., 2005, 2007) was found in the permanently anoxic zone of Lake Pavin (Lepère et al., 2016). Methane is produced in the anoxic zone of the lake though mainly in the sediments, most of it not reaching the atmosphere, as a consequence of being oxidized mainly by aerobic methanotrophs (Lopes et al., 2011), such as *Methylobacter* (Biderre-Petit et al., 2011b).

Although Lake Pavin has been considered as an Archean ocean analog, phototrophic iron oxidation has not been studied in detail in this lake. It is expected, however, that photoferrotrophy is of negligible importance due to the relatively great depth of the chemocline. Instead, most research on microbial mediated Fe-transformations in this lake has focused on facultative (e.g., fermentative) iron-reducing microorganisms (e.g., Lehours et al., 2009, 2010).

### Lake Matano

The deep (590 m) stratified Lake Matano, being the largest, deepest, and oldest ferruginous basin known on Earth (Crowe et al., 2014a), was the first modern Archean ocean analog extensively studied as a possible environment where photoferrotrophy could occur (Crowe et al., 2008a). This tectonic lake, which covers 164 km^2^, is the headwater lake of the five morphologically diverse Malili lakes located on Sulawesi Island, Indonesia. Lake Matano and the even larger Lake Towuti, whose ferruginous sediments have also been recently studied (Vuillemin et al., 2016), are the biggest lakes in this lacustrine district. Despite weak temperature and vertical salinity gradients, stratification persisted over centuries in Lake Matano, aided by the low temperature fluctuations in this equatorial area during the year, and by the steep morphometry of the lake basin. Water renewal in the monimolimnion is estimated to take several hundred years (Katsev et al., 2010, 2017). A quasi-permanent deep pycnocline (and chemocline) located at about 100–120 m separates an oxic upper layer from bottom waters, which are poor in sulfur but rich in methane and reduced iron, with Fe(II) originating from the Fe-rich soils in the catchment (Crowe, 2008).

Phosphorus limitation controls primary production in the oxic layers of Lake Matano (Crowe et al., 2008b), which allows light penetration to the chemocline at 100 m depth, where reduced iron is available for a potential photoferrotrophic activity. Low-light adapted GSB containing bacteriochlorophyll-*e* thrive in the photic chemocline of Lake Matano. Initial calculations based on sulfide availability suggested that the population densities of GSB could not be maintained by sulfide-dependent anoxygenic photosynthesis alone. Accordingly, Crowe et al. (2008a) proposed that a photoferrotrophic metabolism was implied to sustain their growth. However, GSB in Lake Matano are light limited and direct evidence for a photoferrotrophic activity could not yet be provided. Instead, more recent estimations (Crowe et al., 2014a) showed that the “slow growth and C-fixation rates suggest that the Lake Matano GSB can be supported by sulfide even though it only accumulates to scarcely detectable concentrations.” Moreover, barcoding community data demonstrate that GSB in this lake are related to known sulfide-oxidizing phototrophs (Bryant et al., 2012) rather than to Fe(II)-oxidizing GSB. Since either photoheterotrophic growth or the use of H_2_ as an electron donor could also support growth additionally to that provided by sulfide- or iron-driven anoxygenic photosynthesis, the possible occurrence of photoferrotrophy in the water column of Lake Matano would require stronger evidences.

Although apparently lower than previously reported (e.g., Crowe et al., 2011), active methanogenic degradation of organic matter occurs in Lake Matano, causing high methane accumulation in the anoxic bottom waters (Crowe et al., 2011). Even though, recent modeling (Kuntz et al., 2015) estimated that most organic carbon sinking to deep layers is buried in the sediments, ca. 9% is estimated to be degraded via methanogenesis and less than 3% could be degraded by anoxic ferric iron respiration. Part of the methane produced by both acetoclastic and hydrogenotrophic methanogenens (dominated by members of the order *Methanomicrobiales*; Crowe et al., 2011) is oxidized at the chemocline. Meanwhile, anaerobic methane oxidation may be coupled to the reduction of Fe, and/or Mn (Jones et al., 2011) (hydr)oxides or nitrogen oxides (Sturm et al., 2016). As in the ancient oceans, the possible co-occurrence of photoferrotrophy, methanogenesis and iron-mediated anaerobic methane oxidation, establishes Lake Matano as a modern analog potentially harboring the main microbial metabolisms that were driving life in ancient Earth.

### Lake La Cruz

**FIGURE 3:**
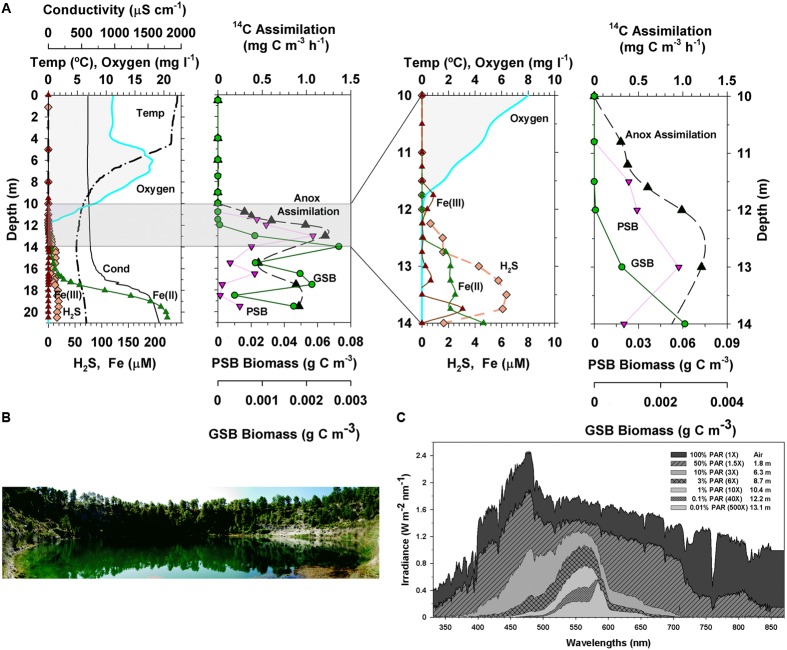
**(A)** Physical and chemical features, anoxygenic phototrophic bacteria biomass, and anoxygenic inorganic carbon assimilation in the vertical profile of Lake La Cruz. The two charts on the right correspond to a zoom of the grey area of those on the left (symbols and lines used are the same for the same variable) **(B)** Photograph of Lake La Cruz and **(C)** light spectral penetration at various depths of the water column. Redrawn from Picazo (2016).

Lake La Cruz (Laguna de la Cruz) is an iron-rich, biogenic meromictic lake located in the karstic system of Cañada del Hoyo (Cuenca, Central-Eastern Spain). The lake became meromictic around 1660 (Julià et al., 1998; Romero-Viana et al., 2010) and is located in a doline (sinkhole) with steep walls protecting the lake from winds. The lake is small, with a surface area of 0.017 km^2^, a maximum diameter of 136 m, and a maximum depth of 21 m. A monimolimnion rich in Ca^2+^, and Fe^2+^, as well as in HCO_3_^-^, CO_2_ and CH_4_, is located in the deepest part of the lake, below a permanent chemocline starting at around 16 m. A seasonal thermal stratification develops in the warmest period and a temporary chemocline then appears from late spring to early summer above the permanent chemocline (Rodrigo et al., 2001). Steep chemical gradients are found both at the bottom of the metalimnion (seasonally) and, permanently, at the stable chemocline (which extends from 16 to 18 m) that separates the mixolimnion from the monimolimnion (**Figure 3**). The very low sulfide concentrations (<0.2 μmol l^-1^ at the chemocline, Oswald et al., 2016) compared to the high concentrations of dissolved reduced iron in the hypolimnion and the monimolimnion makes Lake La Cruz a potential Archean ocean analog were photoferrotrophy could occur.

Surface waters of Lake La Cruz are depleted in nutrients as stratification advances, then phytoplankton becomes nutrient limited, thus increasing water transparency (Picazo, 2016). Light penetrates selectively to deep layers, where sharply stratified planktonic populations of primary producers develop along the physical and chemical gradients of the water column (**Figure 3**). In the oxic and microaerobic layers the photosynthetic planktonic assemblage is dominated by phycoerythrin-containing picocyanobacteria, resulting in a deep chlorophyll maximum at the bottom of the metalimnion during stratification (Camacho et al., 2003; Camacho, 2006), where light availability is around 1% of surface irradiance. Photosynthetic sulfur bacteria, both purple and green, thrive deeper within the anoxic iron-rich, sulfide-poor, waters of the hypolimnion and the monimolimnion (Rodrigo et al., 2000; Romero et al., 2006) (**Figure 3**), being dominated by the PSB *Lamprocystis purpurea* and the GSB *Chlorobium clathratiforme* (Casamayor et al., 2012), with the concomitant presence of *Chlorobium ferrooxidans* (Walter et al., 2014). Chemolithoautotrophic bacteria, including some linked to the iron cycle (Walter, 2011), support important rates of dark inorganic carbon fixation that even exceed the contribution of anoxygenic photosynthesis to total carbon fixation in the lake (Picazo, 2016). Nevertheless, inorganic carbon fixation in the lake is by far dominated by oxygenic photosynthesis, with the highest rates of inorganic carbon fixation (**Figure 3**) occurring at the oxic-anoxic interface during the thermal stratification period (Camacho et al., 2017).

**FIGURE 4:**
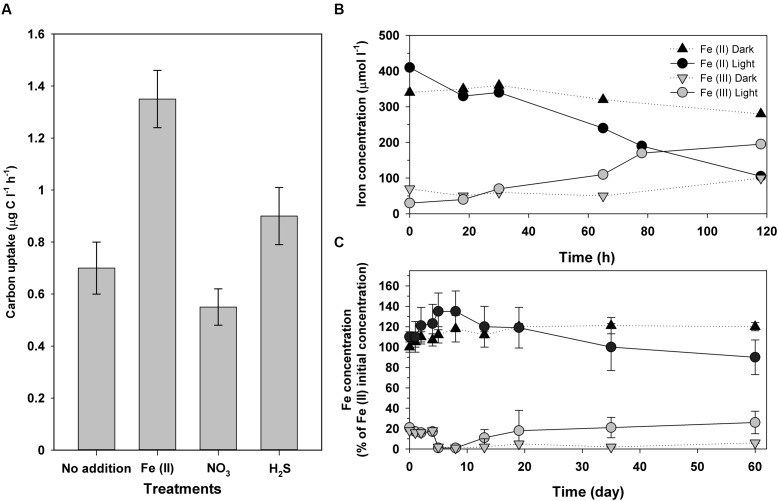
**(A)** *In situ* anoxygenic phototrophic (DCMU amended batches) inorganic carbon uptake in samples from the chemocline of Lake La Cruz amended with Fe(II), NO_3_^-^, and H_2_S, respectively. **(B)** Iron oxidation in samples from Lake La Cruz chemocline, spiked with Fe(II) and DCMU, and incubated under anoxic conditions either in the light or in the dark in a climatic chamber under controlled conditions. **(C)** Iron oxidation of an enrichment culture from Lake La Cruz chemocline, predominantly consisting of GSB closely related to *Chlorobium ferrooxidans*, incubated under anoxic Fe(II)-amended conditions either in the light or in the dark. Modified from Walter et al. (2014)

Fe(III) has low concentrations along the water column of Lake La Cruz. Soluble Fe(II), however, is much more abundant in the anoxic waters, being oxidized in both the seasonal and the permanent chemoclines (**Figure 3**). Aside of the possible chemical reaction with photosynthetically produced O_2_ and the oxidation by microaerophilic chemotrophs in the upper part of the chemocline, photoferrotrophy likely occurs in anoxic waters. Walter et al. (2014) demonstrated that, in the anoxic photic zone, *in situ* inorganic carbon photoassimilation (measured in presence of DCMU, an inhibitor of the oxygenic photosynthesis thus avoiding oxygen release) was significantly higher in Fe(II) amended additions compared to non-amended batches and with those where sulfide or nitrate were added (**Figure 4A**). This was observed only in the presence of light, which supports that photoferroautotrophic processes do occur. Moreover, longer incubations of these anoxic samples in a climatic chamber with Fe(II) additions and DCMU also resulted in Fe(II) oxidation only when exposed to light (**Figure 4B**). Similarly, an enrichment culture of *Chlorobium ferrooxidans* showed the same behavior (**Figure 4C**). The occurrence of light-dependent anaerobic oxidation of Fe(II) in all these experiments represented the first consistent demonstration of photoferrotrophic activity at the chemocline of a modern Archean ocean analog. However, in Lake La Cruz photoferrotrophs (*C. ferrooxidans)* represent only a minor fraction of the anoxygenic phototrophic population, with the majority apparently thriving on sulfur cycling, despite the very low sulfur content in the ferruginous anoxic waters of the photic zone. This majority could potentially follow a similar metabolic path as that described in Lake Matano (Crowe et al., 2014a), within a cryptic sulfur cycle supported by the low concentrations of sulfide available at the chemocline where the low light availability could be co-limiting growth of photosynthetic sulfur bacteria. Additionally, nitrate-reducing Fe(II)-oxidizers have also been found to be active at the chemocline, where they coexist with potential competitors, such as the photoferrotrophs who also utilize reduced iron, as well as with potentially syntrophic organisms (Walter, 2011). The coexistence of phototrophic and nitrate-reducing Fe(II)-oxidizers could be explained by a day-night niche separation with only nitrate-reducing Fe(II)-oxidizers oxidizing Fe(II) in darkness and phototrophs dominating Fe(II) oxidation in daylight (Melton et al., 2012).

**FIGURE 5:**
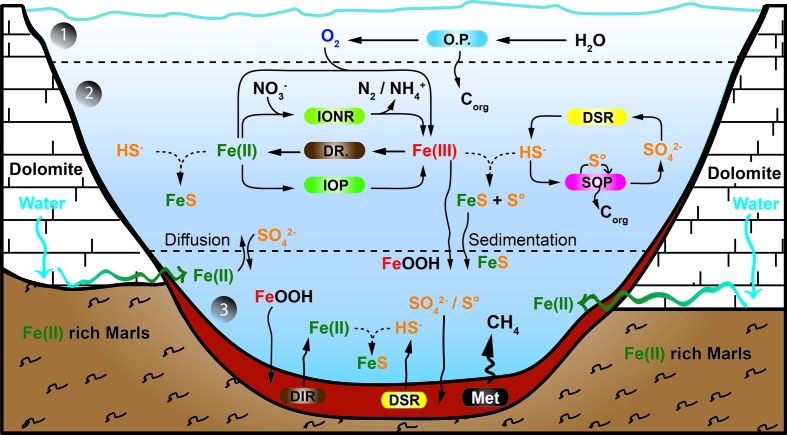
**Model of the iron cycle in Lake La Cruz**. Integration of the iron biogeochemical cycle with the oxygen and the sulfur cycle. Below the epilimnion and metalimnion (1), the chemocline compartment (2) could be an analog to a Neoarchean ferruginous open ocean. However, as neither the deep monimolimnion nor the sediment compartment (3) demonstrate any Fe(III) accumulation it could also be an analog to the euxinic ocean margins characterizing the Neoarchean ocean. All processes from compartment (2) are established along the chemocline. The boundary between compartment (2) and (3) is conceptual and would be situated few centimeters above the sediment. Regular lines indicate biological processes, curved arrows illustrate diffusion/sedimentation processes and broken lines represent chemical processes. The Sediment compartment accumulates sulfur compounds as FeS_am_. (OP) stands for oxygenic phototrophs; (IONR) stands for Fe(II)-oxidizing nitrate-reducing chemotrophs; (DIR) stands for dissimilatory Fe(III)-reducing organotrophs; (IOP) stands for Fe(II)-oxidizing phototrophs; (DSR) stands for dissimilatory sulfate reducing organotrophs; (SOP) stands for sulfide oxidizing phototrophs; and (Met) stands for methanogens.

The major biogeochemical processes operating in the lake are summarized in **Figure 5**. The presented model integrates the iron, carbon, sulfur, and oxygen microbial cycles, including the metabolisms thought to have existed in the late Archean ocean (Walter, 2011). Lake La Cruz thus represents an analog of the late Archean ocean, with oxygenated surface layers hosting oxygenic photosynthesis, overlying a ferruginous anoxic water column holding anoxygenic sulfide-dependent photosynthesis and anoxygenic photoferrotrophy, and with organic sediments where methanogenesis is the main organic mineralization pathway.

### Lake Kivu

Lake Kivu (Descy et al., 2012) is a large (2370 km^2^), deep (485 m), tropical meromictic lake located between the Democratic Republic of the Congo and Rwanda. This lake, of tectonic origin, is the highest of the East African Rift Lakes. This is a very active geological volcanic area with hydrothermal influences on lake deep water layers, which slightly disturb the meromictic conditions (Katsev et al., 2017) created by the vertical salinity gradient originated by salt inputs coming from subaquatic groundwater discharges (Schmid and Wüest, 2012; Ross et al., 2015). The mixolimnion is separated from the monimolimnion, where hydrogen sulfide accumulates to concentrations of ca. 0.3 mM (Pasche et al., 2011; Llirós et al., 2015), by a permanent chemocline located at ∼65 m (Llirós et al., 2012). Large amounts of methane and CO_2_ accumulate in the deep monimolimnion, with the corresponding risk of limnic gas eruptions (Zhang and Kling, 2006; Hirslund, 2012). However, methanotrophs, mainly close relatives of type X CH_4_-oxidizing bacteria (Pasche et al., 2011), very actively oxidize methane (Morana et al., 2015b), mostly aerobically in the oxycline, driving low methane release to the atmosphere (Morana et al., 2015a; Zigah et al., 2015). Nutrient availability in upper layers is low, with average mixolimnetic chlorophyll-a concentrations of 2.2 mg m^-3^. Phytoplankton is dominated by diatoms during the dry season and by cyanobacteria, mainly phycoerythrin-rich picocyanobacteria (Sarmento et al., 2008) during the rainy season, with total phytoplankton biomass of 143–278 g C m^-2^ y^-1^ (Darchambeau et al., 2014). The microbial communities of Lake Kivu have been studied in several basins of the lake (Llirós et al., 2010, 2012, 2015; Plasencia et al., 2010), and their stratified distribution suggest well-defined functional specialization (Inceoğlu et al., 2015a), with highest microbial richness in the anoxic zone (Inceoğlu et al., 2015b). Brown-colored species of GSB permanently developed at 11 m depth in Kabuno Bay and sporadically in the anoxic waters of the main basin (Llirós et al., 2012, 2015).

Kabuno Bay, where *in situ* photoferrotrophy has been reported (Llirós et al., 2015), is separated from the main basin by a shallow (∼7 m) volcanic sill that restricts water exchange with the main basin. Kabuno Bay, with a maximum depth of 150 m, has a steep pycnocline around 10 m depth (Katsev et al., 2014), with a very narrow oxycline and a ferruginous water column below with up to 1.2 mM of Fe(II) deriving from hydrothermal sublacustrine sources (Llirós et al., 2015). Light penetrates well into the Fe(II)-rich, sulfide poor (0.6 μM maximum in the illuminated chemocline), anoxic waters, with GSB comprising up to 30% of the total microbial community in the chemocline. Anoxygenic phototrophs also include PSB and *Chloroflexi*, though being less abundant. The Bchl-*e*-containing GSB were isolated and sequenced, being closely related to *Chlorobium ferrooxidans* strain KoFox (DSM strain 13031). Anoxygenic phototrophs largely dominated CO_2_ fixation in the illuminated redoxcline of Kabuno Bay (Morana et al., 2016). Light-dependent Fe(II) oxidation rates of up to 100 μmol Fe l^-1^ d^-1^ have been reported (Llirós et al., 2015) at the chemocline, much higher than those reported for lakes Matano and La Cruz (**Table 1**), and the photoferrotrophic contribution to CO_2_ fixation in the Kabuno Bay of Lake Kivu by these anoxygenic phototrophs was significant. A tightly coupled pelagic Fe-oxidation-reduction cycle was observed, with much higher rates than those of sulfate reduction and potential sulfide oxidation (Llirós et al., 2015). Actually, rates of photoferrotrophy in the Kabuno Bay water column (3.4 mol C m^-2^ yr^-1^) are within the range of those modeled for global photoferrotrophic production in Earth’s early ferruginous oceans (1.4 mol C m^-2^ yr^-1^) (Llirós et al., 2015). Contrasting to *C. ferrooxidans* KoFox, the Kabuno Bay GSB isolate appears to be specifically adapted to the pelagic habitat, contains Bchl-*e* (conferring low-light adaptation) instead of Bchl-*c*, and grows in pure culture instead of in co-culture. Incubation experiments with the Kabuno Bay isolate also demonstrate its capacity to grow photoferrotrophically under very low light conditions (i.e., 0.64 μ E m^-2^ s^-1^) apparently oxidizing Fe(II) at a rate of 1.4 mmol l^-1^ d^-1^ (Llirós et al., 2015).

## Some Concluding Remarks and Perspectives

Our knowledge on photoferrotrophy and its past and current significance has tremendously increased in the last decade. We now have evidence from pure cultures as well as from environmental studies that this metabolism could have been a significant process in the early times of our planet, but also that it remains as a relict example of how this primitive Earth may have been functioning during hundreds of millions years. These advances likely place photoferrotrophy among the oldest photosynthetic processes on Earth, probably playing an important biogeochemical role during most of the Archean Eon. It has also been demonstrated that oceans evolved from predominantly reduced to the current oxidized conditions. Interestingly, these primordial anoxic ferruginous conditions still prevail in environments such as the iron-rich meromictic lakes. The study of these modern analogs of primitive oceans has revealed that in many of them GSB related to *Chlorobium ferrooxidans* are the main photoferrotrophs, but also that the current ecological and biogeochemical significance of photoferrotrophy is minor. Moreover, even when photoferrotrophs are abundant in an anoxic, iron rich, natural environment, sulfur driven anoxygenic photosynthesis still plays an important or dominant role despite the typically very low sulfide concentrations in ferruginous systems. This, in addition to the fact that bacterial anoxygenic photosynthesis is restricted to small compartments of the modern biosphere, implies that the current ecological relevance of Fe(II)-driven anoxygenic photosynthesis is circumstantial. Exploring photoferrotrophy, however, provides insights into how photosynthesis appeared on Earth and how it progressively shaped the biogeochemistry of our planet.

## Author Contributions

AC, JZ, and XW conceived and designed the work. All coauthors provided information and data to the manuscript. AC made the extensive critical review and wrote the manuscript. XW and AP made the figures. JZ, XW, and AP critically revised the manuscript and provided parts of the text. AC prepared and submitted the final version of the manuscript.

## Conflict of Interest Statement

The authors declare that the research was conducted in the absence of any commercial or financial relationships that could be construed as a potential conflict of interest.
